# Changes to the US Preventive Services Task Force Screening Guidelines and Incidence of Breast Cancer

**DOI:** 10.1001/jamanetworkopen.2024.52688

**Published:** 2024-12-27

**Authors:** Carina Zhang-Petersen, Michelle Sowden, Jennifer Chen, Julia Burns, Brian L. Sprague

**Affiliations:** 1 Department of Surgery, University of Vermont, Burlington; 2 University of Vermont Cancer Center, Burlington; 3 Larner College of Medicine, University of Vermont, Burlington

## Abstract

**Importance:**

The 2009 US Preventive Services Task Force breast cancer screening guideline changes led to decreases in screening mammography, raising concern about potential increases in late-stage disease and more invasive surgical treatments.

**Objective:**

To investigate the incidence of breast cancer by stage at diagnosis and surgical treatment before and after the 2009 guideline changes.

**Design, Setting, and Participants:**

This population-based, epidemiologic cohort study of women aged 40 years or older used 2004 to 2019 data from the National Cancer Institute’s Surveillance, Epidemiology, and End Results Program. Age- and stage-specific breast cancer incidence rates and the proportion of breast cancers treated by partial mastectomy, total mastectomy, and total mastectomy with reconstruction were calculated. Data analyses were conducted from August 2023 to February 2024.

**Interventions or Exposures:**

Age group (40-49, 50-74, and ≥75 years).

**Main Outcomes and Measures:**

Annual percent changes (APCs) in stage-specific breast cancer incidence and proportions of cases treated with each surgery type.

**Results:**

This cohort study included 2 022 250 women (354 263 [17.5%] aged 40-49 years, 1 279 542 [63.2%] aged 50-74 years, and 388 445 [19.2%] aged ≥75 age group, from a total of 2 023 541 women) diagnosed with breast cancer. Rates of in situ breast cancer decreased since 2009 (eg, APC, –0.69 [95% CI, –2.77 to –0.18] for women aged 50-74 years). Localized breast cancer rates increased steadily during 2004 to 2019 in women aged 40 to 74 years (eg, APC, 1.18 [95% CI, 1.02-1.34] for women aged 50-74 years), with no evidence of a change in trend during the study period. Regional cancer rates decreased or did not change. Distant cancer rates were flat since 2012 among women aged 40 to 74 years and increased steadily for those 75 years or older during 2004 to 2019 (APC, 1.40 [95% CI, 1.00-1.82]). The proportion of cases treated with partial mastectomy decreased during 2004 to 2012 (eg, APC, –0.77 [95% CI, –2.96 to –0.03] among women aged 50-74 years with localized cancer), whereas the proportion of cases treated with total mastectomy with reconstruction increased (eg, APC, 20.17 [95% CI, 16.50-33.16]). During 2012 to 2019, the proportion of cases treated with total mastectomy decreased (eg, APC, –2.44 [95% CI, –3.45 to –1.61] for women aged 50-74 years with localized cancer), and the proportion of cases treated with partial mastectomy increased (eg, APC, 1.70 [95% CI, 0.90-4.08] for women aged 50-74 years).

**Conclusions and Relevance:**

In this cohort study, in situ breast cancer decreased since 2009, consistent with decreasing use of screening mammography since the 2009 guideline changes, but this decrease did not appear to have translated to more advanced breast cancer stages at diagnosis or decreases in the proportion of cases treated with partial mastectomy. Further research is needed to understand the long-standing increase in localized invasive breast cancer and the decrease in regional invasive breast cancer observed during the past 20 years in the context of decreased breast cancer screening.

## Key Points

**Question:**

How have trends in breast cancer stage at diagnosis and surgical management changed since the controversial changes to the US Preventive Services Task Force mammography screening guidelines in 2009?

**Findings:**

This cohort study of 2 022 250 women with breast cancer demonstrated that in situ breast cancer incidence decreased among women aged 40 years or older since 2009, whereas findings for other breast cancer stages and surgical treatment did not correlate with the 2009 guideline changes.

**Meaning:**

Although rates of in situ breast cancer have decreased since 2009, there did not appear to be evidence that this has translated to more advanced breast cancer incidence or more invasive operations.

## Teaser

This cohort study of women with breast cancer investigates cancer stage at diagnosis and surgical treatment before and after the 2009 US Preventive Services Task Force (USPSTF) guideline changes.

## Introduction

In 2009, the US Preventive Services Task Force (USPSTF) made controversial changes to their breast cancer screening guidelines for women at average risk of developing breast cancer.^1^ Before this change, women older than 40 years were recommended to undergo screening mammography every 1 to 2 years.^2^ The 2009 changes recommended an individualized decision to undergo biennial screening for women aged 40 to 49 years, routine biennial screening for women aged 50 to 74 years, and no screening recommendation (insufficient evidence) for women older than 75 years.^1^ These changes were recommended to minimize psychological distress, false-positive imaging results and biopsy recommendations, and overdiagnosis.

Since 2009, numerous publications^3,4,5,6^ have reported reductions in the use of breast cancer screening in the US. Decreases in screening use raise concern about potential increases in late-stage disease. Some studies^7,8^ have shown that women diagnosed with breast cancer on biennial, as opposed to annual, mammographic screening have more poor prognostic factors, later-stage cancers, larger tumor sizes, and more interval cancers. However, few studies to date have reported changes in stage distribution or the incidence of regional or advanced cancers after the 2009 screening guidelines. If delays in diagnoses due to less intensive screening result in larger tumor sizes,^7,8^ this could also lead to more extensive surgical management, particularly among women with larger tumors, which if diagnosed sooner could have been eligible for breast-conserving therapy as opposed to a total mastectomy.^9^ We sought to investigate trends in breast cancer stage at diagnosis and surgical treatment before and after the 2009 USPSTF guideline changes.

## Methods

### Study Population

This retrospective cohort study used the National Cancer Institute’s Surveillance, Epidemiology, and End Results Program (SEER)^10^ to obtain breast cancer incidence data. The SEER 22 and SEER 17 registries used in this study include cancer data from 22 and 17 regional US cancer registries, respectively. The SEER 22 registries encompass approximately 47.9% of the US population; the SEER 17 registries are a subset encompassing approximately 26.5% of the US population.^10^ Our study was restricted to women diagnosed with primary breast cancer at 40 years or older from 2004 to 2019 to include 5 years of data before the 2009 guideline change and ended before the impact of the COVID-19 pandemic. No other exclusion criteria were applied. We did not collect race or ethnicity information for this study because we were not aiming to analyze the results according to race or ethnicity. Our analysis was determined to be exempt from review by the University of Vermont Institutional Review Board because the data are publicly available. Informed consent was waived by the University of Vermont Institutional Review Board because data were deidentified. We followed the Strengthening the Reporting of Observational Studies in Epidemiology (STROBE) reporting guidelines.

### Data Collection

Stage-specific breast cancer diagnosis counts were obtained from the SEER 22 registries from 2004 to 2019. Stage at diagnosis was classified using the SEER Summary Stage variable as in situ (noninvasive, ductal, or lobular), localized (confined to breast tissue), regional (lymph node involvement or direct extension to pectoral muscle, chest wall, or skin), or distant (metastatic). For analyses of surgical treatments, we obtained counts of cases treated with partial mastectomy, total mastectomy, and total mastectomy with reconstruction for in situ, localized, and regional diagnoses from the SEER 17 registries because they were unavailable for all study years in the SEER 22 registries. Partial mastectomies (also known as lumpectomy or breast-conserving surgery) included partial mastectomies with or without nipple resection and reexcision for gross or microscopic residual disease. Total mastectomies included those with or without removal of an uninvolved contralateral breast. Total mastectomy with reconstruction included those performed unilaterally or bilaterally with an uninvolved contralateral breast, a nipple-sparing mastectomy, and reconstruction using tissue, implant, or a combination.

Patients who did not undergo surgery or underwent an unknown type of surgery were excluded from the analyses of surgical trends. Patients aged 75 years or older who underwent total mastectomy with reconstruction were also excluded because there were few in this age group who underwent reconstruction. The standard treatment for distant breast cancer is systemic therapy^11^; thus, distant cancers were excluded from the analysis of surgical trends.

### Statistical Analysis

Data analyses were conducted from August 2023 to February 2024. Stage-specific breast cancer incidence rates were calculated by age (40-49, 50-74, and ≥75 years) using the diagnosis counts and the corresponding US Census female population denominators available in SEER. Incidence rates for the 50-to-74-year age group were age adjusted to the 2000 US Standard Population because this age grouping covers a wide range of ages. These incidence rates were then analyzed using Joinpoint Trend Analysis Software,^12^ which calculated annual percent changes (APCs) with 95% CIs and 2-sided tests for statistical significance for each stage and age group.

We calculated the proportion of in situ, localized, and regional breast cancers treated with partial mastectomy, total mastectomy, and total mastectomy with reconstruction. Trends in these proportions were analyzed using Joinpoint Trend Analysis to calculate APCs with 95% CIs and 2-sided tests for statistical significance for the surgery types, stage, and age groups described.

## Results

The study included 2 022 250 women aged 40 years or older (354 263 [17.5%] aged 40-49 years, 1 279 542 [63.2%] aged 50-74 years, and 388 445 [19.2%] aged ≥75 age group, from a total of 2 023 541 women) diagnosed with breast cancer reported by the SEER cancer registries during 2004 to 2019.

### Trends in Stage at Diagnosis

#### Ages 40 to 49 Years

**Figure 1.**
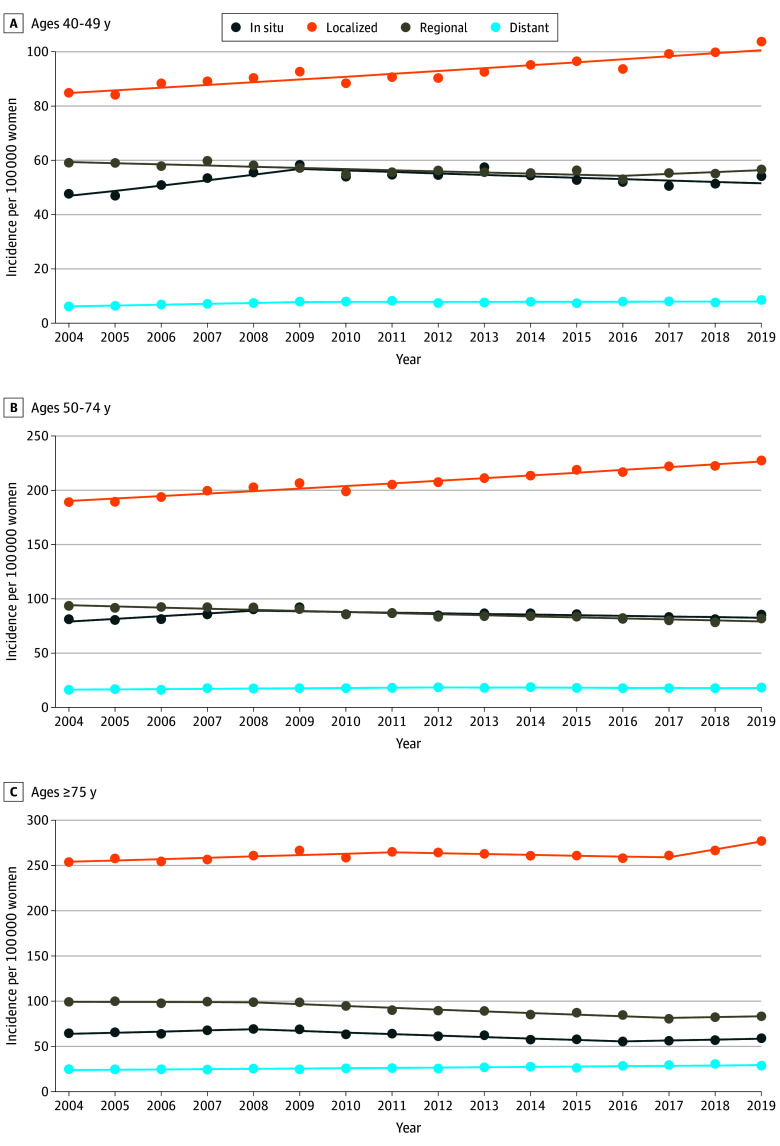
Breast Cancer Incidence by Age at Diagnosis Within the Surveillance, Epidemiology, and End Results 21 Registries, 2004-2019 Trend lines represent Joinpoint modeled rates.

In women aged 40 to 49 years, rates of in situ breast cancer increased before 2009 (APC, 3.91 [95% CI, 1.97-7.74]) (Figure 1A; eTable 1 in Supplement 1). Since 2009, there was a decrease in rates of in situ breast cancer (APC, −0.97 [95% CI, −2.10 to −0.28]). Rates of localized cancers steadily increased from 2004 to 2019 (APC, 1.14 [95% CI, 0.82-1.47]). Rates of regional cancers decreased during 2004 to 2016 (APC, −0.75 [95% CI, −1.72 to −0.50]), with no significant change after 2016. Before 2009, rates of distant cancers increased (APC, 4.78 [95% CI, 2.74-9.52]), followed by no significant change since 2009.

#### Ages 50 to 74 Years

In women aged 50 to 74 years, rates of in situ breast cancer increased during 2004 to 2008 (APC, 3.05 [95% CI, 0.48-11.92]) (Figure 1B; eTable 1 in Supplement 1). Since 2008, there was a decrease in rates of in situ breast cancer (APC, −0.69 [95% CI, −2.77 to −0.18]). Rates of localized cancers steadily increased from 2004 to 2019 (APC, 1.18 [95% CI, 1.02-1.34]). Rates of regional cancers steadily decreased (APC, −1.14 [95% CI, −1.42 to −0.87]) from 2004 to 2019. Distant cancers increased from 2004 to 2012 (APC, 1.43 [95% CI, 0.81-4.04]), followed by no significant change since 2012.

#### Ages 75 Years or Older

In women aged 75 years or older, rates of in situ breast cancer increased during 2004 to 2008 (APC, 1.93 [95% CI, 0.48-4.07]) (Figure 1C; eTable 1 in Supplement 1). From 2008 to 2016, there was a decrease in rates of ductal carcinoma in situ (APC, −2.65 [95% CI, −3.87 to −2.14]). Since 2016, there was no significant change in the incidence of in situ breast cancer. Rates of localized cancers experienced an overall increase (APC, 0.58 [95% CI, 0.30-1.41]) in 2004 to 2011, a decrease (APC, −0.35 [95% CI, −1.36 to −0.03]) in 2011 to 2017, and an increase (APC, 3.34 [95% CI, 1.71-4.53]) in 2017 to 2019. Rates of regional cancers decreased from 2008 to 2017 (APC, −2.11 [95% CI, −4.19 to −1.77]) with no significant change thereafter. There was a steady increase in distant cancers (APC, 1.40 [95% CI, 1.00-1.82]) from 2004 to 2019.

### Surgical Treatment

#### Ages 40 to 49 Years

**Figure 2.**
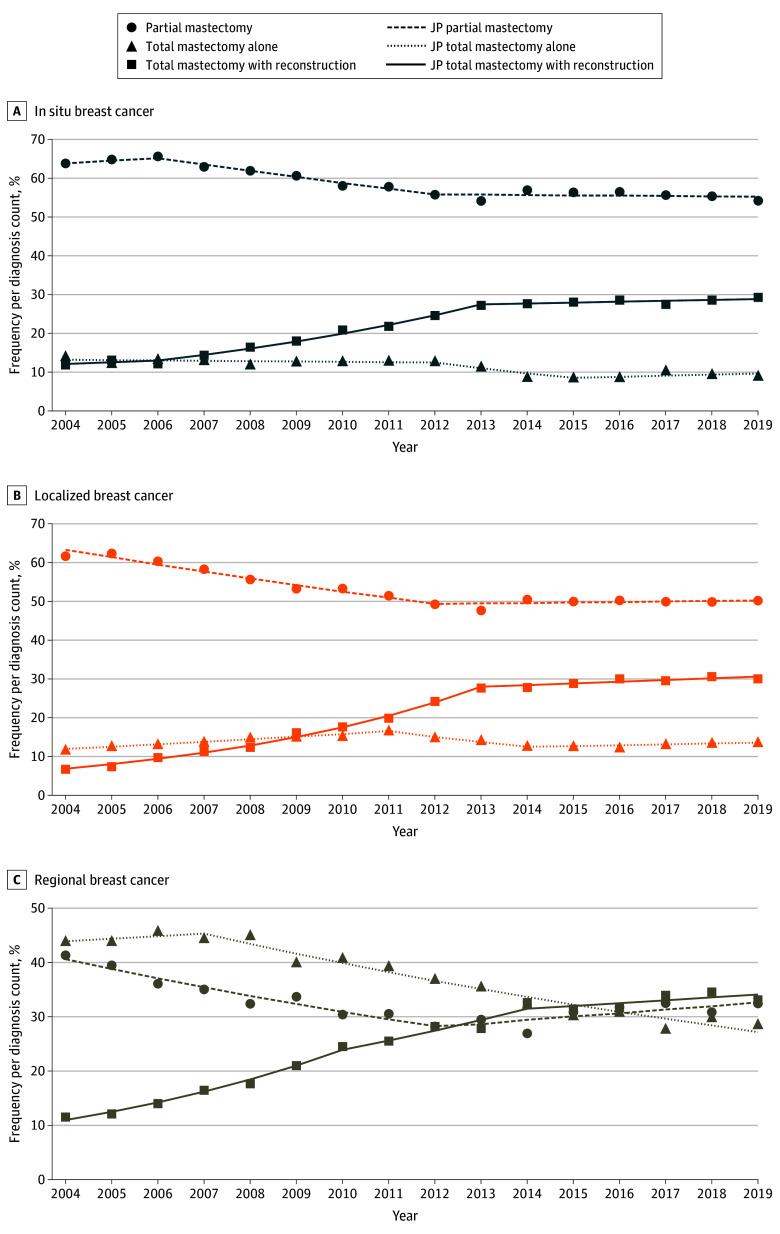
Breast Cancer Surgical Trends by Disease Type Among Women Aged 40 to 49 Years Within the Surveillance, Epidemiology, and End Results 17 Registries, 2004-2019 Trend lines represent Joinpoint (JP) modeled rates.

In women aged 40 to 49 years, there was a trend toward a reduced frequency of partial mastectomies before 2012, an increased frequency of total mastectomies with reconstruction throughout the study period, and a general decrease in total mastectomies alone (Figure 2; eTable 2 in Supplement 1). For example, among women with in situ breast cancer, the frequency of partial mastectomies decreased (APC, −2.54 [95% CI, −3.89 to −2.02]) (Figure 2A) in 2006 to 2012, with a concurrent increase in total mastectomies with reconstruction (APC, 11.31 [95% CI, 0.72-14.08]). From 2012 to 2015, there was a subsequent decrease in total mastectomies alone (APC, −11.87 [95% CI, −15.05 to −6.10]). In women with localized cancers, total mastectomies alone increased until 2011 alongside the increase in total mastectomies with reconstruction. This increase was followed by a decrease until 2014 and a slight increase thereafter (Figure 2B). Regional cancers followed similar trends but experienced an increase in partial mastectomies from 2012 to 2019 and a more prolonged decrease in total mastectomies from 2007 until 2019 (Figure 2C).

#### Ages 50 to 74 Years

**Figure 3.**
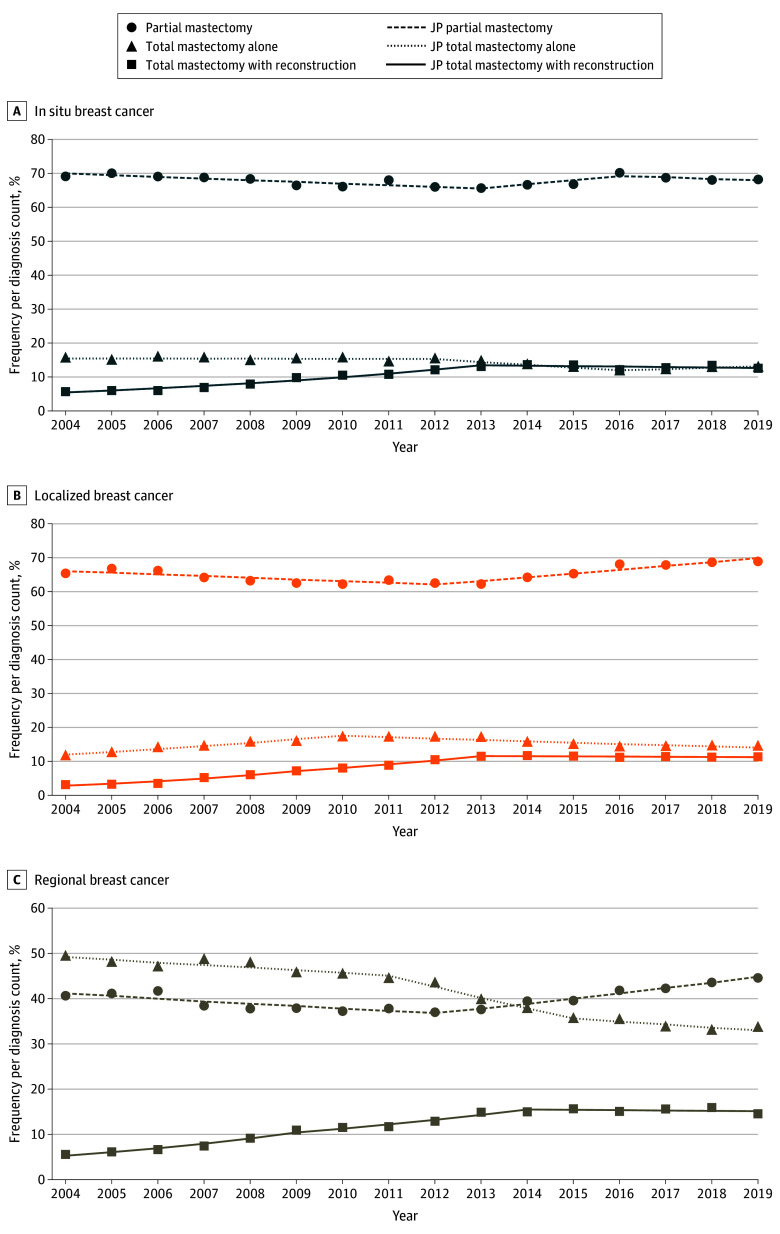
Breast Cancer Surgical Trends by Disease Type Among Women Aged 50 to 74 Years Within the Surveillance, Epidemiology, and End Results 17 Registries, 2004-2019 Trend lines represent Joinpoint (JP) modeled rates.

In women aged 50 to 74 years, there was a decrease in the frequency of partial mastectomies until 2012, with a concurrent increase in the frequency of total mastectomies with reconstruction (Figure 3; eTable 2 in Supplement 1). These trends were followed by a decrease in total mastectomies alone and, for localized and regional cancers, an increase in partial mastectomies. For example, for women diagnosed with localized cancers, there was a decrease in the frequency of partial mastectomies from 2004 to 2012 (APC, −0.77 [95% CI, −2.96 to −0.03]) (Figure 3B), with a concurrent increase in the frequency of both total mastectomies alone (APC, 6.58 [95% CI, 4.69-9.33]) and total mastectomies with reconstruction (APC, 20.17 [95% CI, 16.50-33.16]). Since 2012, there had been an increase in partial mastectomies (APC, 1.70 [95% CI, 0.90-4.08]), with a decrease in total mastectomies alone from 2010 to 2019 (APC, −2.44 [95% CI, −3.45 to −1.61]). There was very little change in the frequency of partial mastectomies among women diagnosed with in situ breast cancer across the study period (Figure 3A). Women diagnosed with in situ breast cancer and regional cancers (Figure 3C) did not experience the increase in total mastectomies alone before 2012 that was seen among women diagnosed with localized cancers.

#### Ages 75 Years or Older

**Figure 4.**
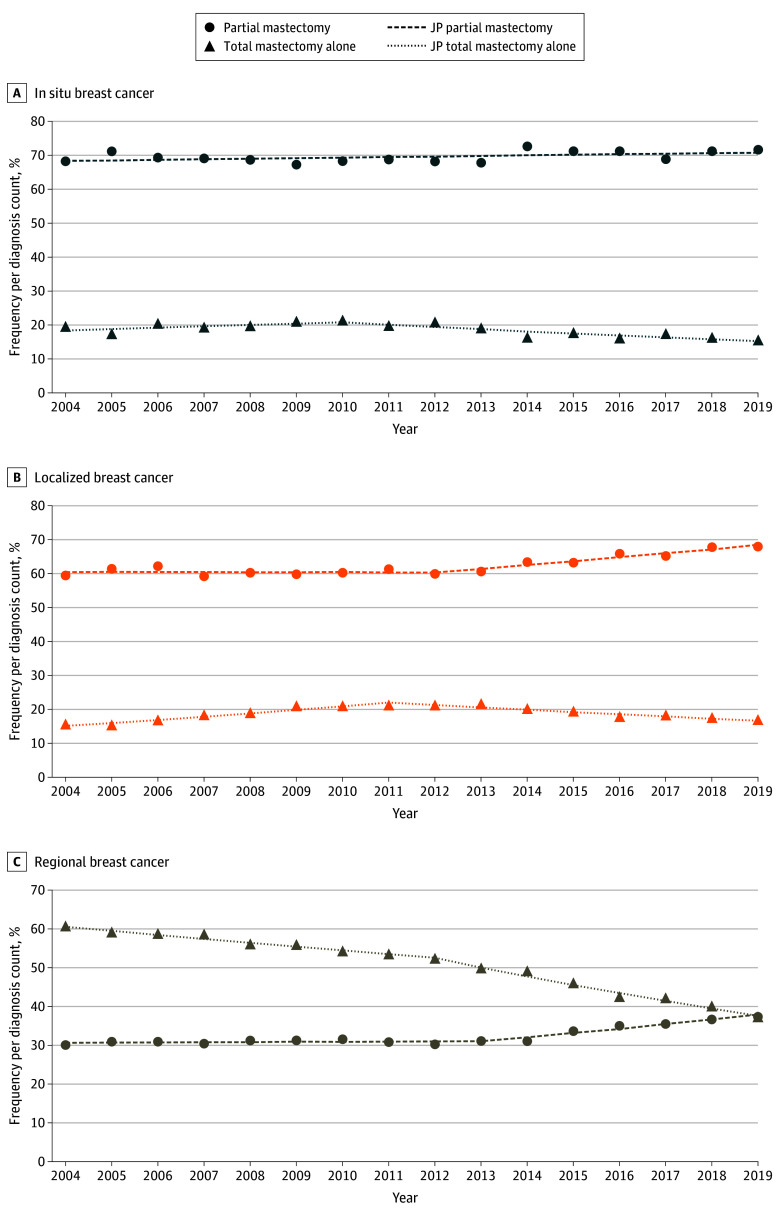
Breast Cancer Surgical Trends by Disease Type Among Women Aged 75 Years or Older Within the Surveillance, Epidemiology, and End Results 17 Registries Trend lines represent Joinpoint (JP) modeled rates.

In women aged 75 years or older, surgical treatment generally trended away from total mastectomy, with an increase in the rates of partial mastectomy (Figure 4; eTable 2 in Supplement 1). For example, the rates of total mastectomy for regional cancers decreased from 2004 to 2012 (APC, −1.77 [95% CI, −2.19 to −1.25]) (Figure 4C) and from 2012 to 2019 (APC, −4.65 [95% CI, −5.40 to −4.11]). Partial mastectomy rates increased from 2013 to 2019 (APC, 3.33 [95% CI, 2.44-4.88]). In women with in situ breast cancer, partial mastectomy rates have remained steady (Figure 4A). In women diagnosed with localized cancers, total mastectomy initially increased from 2004 to 2012. This increase was followed by a decrease in total mastectomies and concurrent increase in partial mastectomies (Figure 4B).

## Discussion

We examined patterns in stage-specific breast cancer incidence and surgical treatments for breast cancer in relation to the timing of the 2009 USPSTF guideline changes. Our results provide evidence of a decrease in in situ breast cancer incidence across all age groups since 2009, consistent with previously reported decreases in the use of breast cancer screening. Patterns in the rates of localized, regional, and distant breast cancer did not change markedly in relation to the timing of the guideline changes. Patterns in the rate of partial vs total mastectomies did not correlate temporally to the timing of the guideline change, and the patterns in surgical treatments after 2012 suggest a trend toward less invasive surgical procedures.

In situ breast cancer is predominantly detected on mammography screening.^13^ The decreases we observed in in situ breast cancer incidence are consistent with the decreases in screening use observed after the 2009 USPSTF screening guideline changes.^3,4,5,6^ With this decrease in in situ breast cancer diagnoses since 2009, we anticipated that a proportion of undetected in situ breast cancer would progress, potentially contributing to an increasing incidence of localized breast cancers starting after 2009. However, we observed no discrete shifts in localized cancer incidence trends after 2009; rather, there was a steady continued increase in localized breast cancer consistent with the pattern observed before 2009. A possible exception was observed in women aged 75 years or older, among whom localized invasive cancer incidence increased beginning in 2017 after a period of decreasing in situ breast cancer incidence and stable localized invasive cancer incidence. However, this would require a long sojourn time (≥9 years) to progression to be attributed to the decrease in in situ breast cancer incidence that began in 2008.

With the substantial decreases in screening in women aged 40 to 49 years,^3,4,5^ we would also anticipate increases in regional and distant cancers in that age group after 2009, which were not seen. There was no evidence for an increase in regional cancers across any age group. Women aged 75 years or older^6^ experienced an increasing incidence of distant stage cancers during the study period, but the trend began in 2004, well before the 2009 guideline changes. Overall, there was no clear evidence that reductions in screening and in situ breast cancer diagnoses translated to an increasing incidence of more advanced-stage disease during the study period.

Regarding surgical treatment, there did not appear to be strong evidence that the 2009 screening guideline changes contributed to more aggressive surgical management for in situ, localized invasive, or regional breast cancer. With less screening, we anticipated larger in situ tumor sizes, leading to more total mastectomies, because larger tumors may render some patients ineligible for breast-conserving therapy. There was a trend toward increased total mastectomy and reconstruction rates in women aged 40 to 74 years, but the temporality does not fit our predictions because this trend began before 2009 and leveled off by 2013. After 2012, rates of total mastectomy mostly decreased, whereas rates of partial mastectomy increased or remained the same across all age groups and stages. This finding suggests that lower rates of screening may not have led to increasing incidence of larger cancers that necessitated total mastectomy. Alternatively, this finding could be explained by other factors influencing surgical management of breast cancer, including the increased use of oncoplastic reductions,^14,15^ which allow for good cosmetic outcomes with larger amounts of breast tissue removed. In addition, the shift toward partial mastectomies is consistent with the more recent deescalation of surgical treatment for older patients because some studies^16,17,18^ found no survival benefit of more intensive treatments as opposed to less invasive options.

We observed a consistent upward trend in total mastectomies with reconstruction vs total mastectomies alone during 2004 to 2013. The cause of this shift toward reconstruction is unclear but could be related to increased awareness or availability of reconstructive options, avoidance of radiation, or the desire to avoid future routine screening mammography. In this study, we were unable to determine the motive for reconstruction—whether patients chose reconstruction for cosmesis despite being eligible for breast-conserving therapy, for desired cancer risk reduction in the contralateral breast because they wanted to avoid radiation therapy, or because their tumor size necessitated a total mastectomy. The literature surrounding the 2004 to 2013 time frame suggests that women undergo total mastectomies because they desire the reconstructive options, even if they are eligible for breast-conserving therapy.^14,19^

We did not evaluate the frequency of axillary surgery because any observed trends would have been heavily influenced by the Z11 trial, which caused a shift away from axillary surgery for women diagnosed with T1 to T2 cancers with 1 to 2 positive sentinel nodes.^20,21^ However, these patients would still undergo a partial vs a total mastectomy independent of axillary nodal status based on tumor characteristics, including size. Thus, the partial mastectomy group included those with and without axillary dissection, the total mastectomy alone group included those who underwent a modified radical mastectomy without reconstruction, and the total mastectomy with reconstruction group included those who underwent a modified radical mastectomy with reconstruction.

### Limitations

Several limitations should be considered in the interpretation of our results. First, the SEER registries provide a large, diverse sample population that broadly represents the country, but they do not capture the entire US population. Second, as an observational, retrospective study, this study could not directly assess the effect of screening use on disease stage at diagnosis or surgical treatment patterns. Other temporal patterns could contribute to the observed patterns and obscure the impact of screening changes. For example, decreases in cancer detection via reduced screening mammography use may have been offset by enhanced cancer detection using digital breast tomosynthesis technology in mammography examinations. Digital breast tomosynthesis has disseminated widely since its approval by the US Food and Drug Administration in 2011^22^ and has elevated cancer detection compared with conventional digital mammography.^23^ In addition, increased uptake of supplemental screening modalities (eg, ultrasonography and magnetic resonance imaging) in response to mandated dense breast disclosures^24,25^ may have also ameliorated the impact of reduced screening mammography use. Similarly, improvements in available surgical treatments (eg, cosmesis after oncoplastic reductions for large amounts of breast tissue removed^14^), increasing use of neoadjuvant chemotherapy, and other factors have likely influenced trends in surgical management. Third, we evaluated patterns in stage at diagnosis as characterized by SEER Summary Stage but did not investigate American Joint Committee on Cancer stage at diagnosis or tumor size and nodal status subcomponents of staging criteria. Fourth, mode of detection is unavailable in this dataset, so we were unable to evaluate screen-detected vs symptom-detected cases. Fifth, our analysis covers a 10-year follow-up period from the 2009 USPSTF guidelines; given the slow progression of some in situ disease, it is possible that increases in invasive breast cancer incidence may occur after 10 years of follow-up.

In April 2024, the USPSTF released updated breast cancer screening guidelines that expanded routine biennial screening to include women aged 40 to 49 years rather than starting at 50 years of age in response to the increase in invasive breast cancer incidence in this age group.^26^ Future studies will be required to evaluate the effect of these new recommendations on breast cancer screening use and incidence patterns.

## Conclusions

In this cohort study, the incidence of in situ breast cancer decreased with the decrease in use of screening mammography dating back to the 2009 USPSTF guideline change. However, to date, there does not appear to be evidence that reductions in screening and in situ breast cancer diagnoses have translated to increases in the incidence of more advanced-stage breast cancer. Since 2012, there has been a shift away from total mastectomies to partial mastectomies, likely reflecting surgical de-escalation literature and the increase in oncoplastic reconstruction options. Although the 2009 guideline changes did not appear to cause an inflection in the incidence of localized, regional, or distant breast cancer, further research is needed to understand the long-standing increase in localized invasive breast cancers and the decrease in regional invasive breast cancers observed during the past 20 years in the context of decreased breast cancer screenings.

## Notes

Supplement 1. **eTable 1**. Joinpoint Analysis of Trends in Breast Cancer Incidence Rates in the U.S., SEER 22 Registries, 2004-2019 **eTable 2**. Joinpoint Analyses of Trends in Breast Cancer Surgeries in the U.S., SEER 17 Registries, 2004-2019

Supplement 2.
Data Sharing Statement
