# Supplementary material for: Changes to the US Preventive Services Task Force Screening Guidelines and Incidence of Breast Cancer
Source: JAMA Netw Open. 2024 Dec 27;7(12):e2452688. doi: 10.1001/jamanetworkopen.2024.52688 (PMC11681378; doi:10.1001/jamanetworkopen.2024.52688)
Supplement: Supplement 1. — eTable 1. Joinpoint Analysis of Trends in Breast Cancer Incidence Rates in the U.S., SEER 22 Registries, 2004-2019 eTable 2. Joinpoint Analyses of Trends in Breast Cancer Surgeries in the U.S., SEER 17 Registries, 2004-2019 [file jamanetwopen-e2452688-s001.pdf]

Supplementary Online Content

Zhang-Petersen C, Sowden M, Chen J, Burns J, Sprague BL. Changes to the US Preventive Services Task Force screening guidelines and incidence of breast cancer. *JAMA Netw Open*. 2024;7(12):e2452688. doi:10.1001/jamanetworkopen.2024.52688

eTable 1. Joinpoint Analysis of Trends in Breast Cancer Incidence Rates in the U.S., SEER 22 Registries, 2004-2019

eTable 2. Joinpoint Analyses of Trends in Breast Cancer Surgeries in the U.S., SEER 17 Registries, 2004-2019

This supplementary material has been provided by the authors to give readers additional information about their work.

© 2024 Zhang-Petersen C et al. *JAMA Network Open.*

eTable 1. Joinpoint analysis of trends in breast cancer incidence rates in the U.S., SEER 22 Registries, 2004-2019.

	Stage at Diagnosis	Year	APC	95% Confidence Interval	Year	APC	95% Confidence Interval	Year	APC	95% Confidence Interval
Ages 40-49 years										
	In Situ	2004-2009	3.91*	1.97 to 7.74	2009-2019	-0.97*	-2.1 to -0.28			
	Localized	2000-2019	1.14*	0.82 to 1.47						
	Regional	2004-2016	-0.75*	-1.72 to -0.5	2016-2019	1.29	-0.38 to 3.77			
	Distant	2004-2009	4.78*	2.74 to 9.52	2009-2019	0.2	-1.03 to 0.91			
	All Stages	2004-2008	1.95*	0.76 to 5.31	2008-2016	-0.14	-2.26 to 0.33	2016-2019	1.84	-0.02 to 4.45
Ages 50-74 years										
	In Situ	2004-2008	3.05*	0.48 to 11.92	2008-2019	-0.69*	-2.77 to -0.18			
	Localized	2004-2019	1.18*	1.02 to 1.34						
	Regional	2004-2019	-1.14*	-1.42 to -0.87						
	Distant	2004-2012	1.43*	0.81 to 4.04	2012-2019	-0.41	-2.72 to 0.29			
	All Stages	2004-2019	0.35*	0.11 to 0.61						
Ages 75+ years										
	In Situ	2004-2008	1.93*	0.48 to 4.07	2008-2016	-2.65*	-3.87 to -2.14	2016-2019	1.71	-0.43 to 4.92
	Localized	2004-2011	0.58*	0.3 to 1.41	2011-2017	-0.35*	-1.36 to -0.03	2017-2019	3.34*	1.71 to 4.53
	Regional	2004-2008	-0.14	-1.28 to 2.75	2008-2017	-2.11*	-4.19 to -1.77	2017-2019	1.07	-1.78 to 3.18
	Distant	2004-2019	1.40*	1 to 1.82						
	All Stages	2004-2009	0.2	-0.27 to 1.47	2009-2017	0.88*	-1.98 to -0.65	2017-2019	2.77*	0.82 to 3.92

*Statistically different from zero at alpha = 0.05 level
Age 50-75 incidence rates are standardized to the age 50-74 2000 U.S. Standard Population in 5-year increments

© 2024 Zhang-Petersen C et al. *JAMA Network Open.*

eTable 2. Joinpoint analyses of trends in breast cancer surgeries in the U.S., SEER 17 Registries, 2004-2019

Ages 40-49 years	Year	APC	95% C.I.	Year	APC	95% C.I.	Year	APC	95% C.I.
In Situ									
Partial Mastectomy	2004-2006	0.97	-1.10 to 2.87	2006-2012	-2.54*	-3.89 to -2.02	2012-2019	-0.16	-0.71 to 0.63
Total Mastectomy Alone	2004-2012	-0.71	-1.95 to 1.66	2012-2015	-11.87*	-15.05 to -6.10	2012-2019	3.08	-0.79 to 11.65
Total Mastectomy with Reconstruction	2004-2006	3.55	-0.15 to 10.71	2006-2013	11.31*	0.72 to 14.08	2013-2019	0.84	-0.59 to 2.39
Localized									
Partial Mastectomy	2004-2012	-3.07*	-3.72 to -2.58	2012-2019	0.25	-0.42 to 1.15			
Total Mastectomy Alone	2004-2011	4.74*	4.07 to 5.53	2011-2014	-8.93*	-10.24 to -6.87	2014-2019	1.66*	0.58 to 3.45
Total Mastectomy with Reconstruction	2004-2013	16.90*	15.46 to 18.61	2013-2019	1.51	-0.10 to 3.31			
Regional									
Partial Mastectomy	2004-2012	-4.45*	-6.24 to -3.32	2012-2019	2.08*	0.39 to 4.92			
Total Mastectomy	2004-2007	1.11	-2.81 to 8.58	2007-2019	-4.17*	-6.45 to -3.64			
Total Mastectomy with Reconstruction	2004-2010	13.85*	5.23 to 28.32	2010-2014	7.12	-2.02 to 20.84	2014-2019	1.61	-6.39 to 7.44
**Ages 50-74 years**	**Year**	**APC**	**95% C.I.**	**Year**	**APC**	**95% C.I.**	**Year**	**APC**	**95% C.I.**
In Situ									
Partial Mastectomy	2004-2013	-0.71	-1.26 to 0.06	2013-2016	1.80	-1.27 to 2.51	2016-2019	-0.59	-2.27 to 0.68
Total Mastectomy Alone	2004-2012	-0.12	-0.83 to 0.84	2012-2016	-5.92*	-8.41 to -4.10	2016-2019	2.81	-0.01 to 7.71
Total Mastectomy with Reconstruction	2004-2013	10.58*	8.89 to 13.61	2013-2019	-1.01	-4.29 to 1.25			
Localized									
Partial Mastectomy	2004-2012	-0.77*	-2.96 to -0.03	2012-2019	1.70*	0.90 to 4.08			
Total Mastectomy Alone	2004-2010	6.58*	4.69 to 9.33	2010-2019	-2.44*	-3.45 to -1.61			
Total Mastectomy with Reconstruction	2004-2009	20.17*	16.5 to 33.16	2009-2013	12.81	-1.71 to 15.50	2013-2019	-0.45	-2.34 to 1.68
Regional									
Partial Mastectomy	2004-2012	-1.40*	-2.29 to -0.74	2012-2019	2.90*	2.12 to 3.95			
Total Mastectomy Alone	2004-2011	-1.24	-2.41 to 0.06	2011-2015	-5.73*	-7.96 to -0.57	2015-2019	-1.91	-4.19 to 1.97
Total Mastectomy with Reconstruction	2004-2009	14.48*	10.35 to 31.77	2009-2014	8.26	-3.98 to 12.12	2014-2019	-0.46	-6.52 to 5.09
**Ages 75+ years**	**Year**	**APC**	**95% C.I.**	**Year**	**APC**	**95% C.I.**	**Year**	**APC**	**95% C.I.**
In Situ									
Partial Mastectomy	2004-2019	0.23	-0.06 to 0.53						
Total Mastectomy Alone	2004-2010	2.05	-0.15 to 7.82	2010-2019	-3.43*	-6.29 to -2.21			

© 2024 Zhang-Petersen C et al. *JAMA Network Open.*

Localized						
Partial Mastectomy	2004-2012	-0.04	-2.36 to 0.58	2012-2019	1.82*	1.10 to 4.27
Total Mastectomy Alone	2004-2011	5.50*	4.31 to 6.85	2011-2019	-3.45*	-4.38 to -2.60
Regional						
Partial Mastectomy	2004-2013	0.16	-0.59 to 0.68	2013-2019	3.33*	2.44 to 4.88
Total Mastectomy Alone	2004-2012	-1.77*	-2.19 to -1.25	2012-2019	-4.65*	-5.40 to -4.11

*Statistically different from zero at alpha = 0.05 level

© 2024 Zhang-Petersen C et al. *JAMA Network Open.*
