# Supplementary material for: Changes to the US Preventive Services Task Force Screening Guidelines and Incidence of Breast Cancer
Source: JAMA Netw Open. 2024 Dec 27;7(12):e2452688. doi: 10.1001/jamanetworkopen.2024.52688 (PMC11681378; doi:10.1001/jamanetworkopen.2024.52688)
Supplement: Supplement 2. — Data Sharing Statement [file jamanetwopen-e2452688-s002.pdf]

Data Sharing Statement

Zhang-Petersen. Changes to the US Preventive Services Task Force Screening Guidelines and Incidence of Breast Cancer. *JAMA Netw Open*. Published December 27, 2024. doi:10.1001/jamanetworkopen.2024.52688

Data
Data available: Yes
Data types: Deidentified participant data
How to access data: https://seer.cancer.gov/data-software
When available: With publication

Supporting Documents
Document types: None

Additional Information
Who can access the data: Data is an online database accessible via the stated URL for anyone requesting the data.
Types of analyses: Data is an online database accessible via the stated URL for any purpose.
Mechanisms of data availability: Data is an online database accessible via the stated URL.
